# The genomic medicine center Karolinska 10-year report on genome sequencing for rare diseases and a strategy for stepwise clinical implementation

**DOI:** 10.1186/s13073-026-01611-3

**Published:** 2026-03-30

**Authors:** Anna Lindstrand, Kristina Lagerstedt-Robinson, Anders Jemt, Malin Kvarnung, Sofia Ygberg, Sofie Vonlanthen, Mikael Oscarson, Daniel Nilsson, Nicole Lesko, Angelo Salazar Mantero, Britt-Marie Anderlid, Henrik Arnell, Cecilia Arthur, Svetlana Bajalica-Lagercrantz, Michela Barbaro, Peter Bergman, Erik Björck, Oda Blomqvist Picard, Helene Bruhn, Jonas Carlsten, Sandrina P. Correia, Karl De Geer, Angelica M. Delgado Vega, Emma Ehn, Jesper Eisfeldt, Marlene Ek, Ingegerd Elvers, Martin Engvall, Christoph Freyer, Sofia Frisk, Caroline Graff, Giedré Grigelioniené, Peter Gustafsson, Anna Hammarsjö, Hafdis T. Helgadottir, Maritta Hellström Pigg, Olivia J. Henry, Moa Hägglund, Erik Iwarsson, Vincent Janvid, Maria Johansson Soller, Leif Sundin, Ekaterina Kuchinskaya, Anders Kämpe, Anna Leinfelt, Agne Liedén, Hillevi Lindelöf, Anna Lyander, Helena Malmgren, Maria Mannila, Per Marits, Karin Naess, Ramprasad Neethiraj, Karl Nyren, Christoforos Pappas, Martin Paucar, Nadja Pekkola Pacheco, Lucia Peña Perez, Maria Pettersson, Peter Pruisscher, Chiara Rasi, Annick Renevey, Sophia Rössner, Ellika Sahlin, Erik Stenund, Tommy Stödberg, Mikael Sundin, Karl Svärd, Bianca Tesi, Emma Tham, Håkan Thonberg, Virpi Töhönen, Malin Ueberschär, Karin Wallander, Eini Westenius, Johanna Winberg, Nerges Winblad, Josephine Wincent, Malin Winerdal, Anna Wredenberg, Anna Zetterlund, Rolf H. Zetterström, Ingegerd Öfverholm, Ann Nordgren, Henrik Stranneheim, Valtteri Wirta, Anna Wedell

**Affiliations:** 1 Department of Clinical Genetics and Genomics, Karolinska University Hospital, Stockholm, Sweden; 2 Department of Molecular Medicine and Surgery, Karolinska Institutet, Stockholm, Sweden; 3 Genomic Medicine Centre Karolinska, Karolinska University Hospital, Stockholm, Sweden; 4 Department of Microbiology, Tumor and Cell Biology, Science for Life Laboratory, Karolinska Institutet, Stockholm, Sweden; 5 Centre for Inherited Metabolic Diseases, Karolinska University Hospital, Stockholm, Sweden; 6 Astrid Lindgren Children’s Hospital, Karolinska University Hospital, Stockholm, Sweden; 7 Department of Clinical Immunology and Transfusion Medicine, Karolinska University Hospital, Stockholm, Sweden; 8 Department of Molecular Medicine and Surgery, Science for Life Laboratory, Karolinska Institutet, Stockholm, Sweden; 9 Department of Women’s and Children’s Health, Karolinska Institutet, Stockholm, Sweden; 10 Department of Oncology-Pathology, Karolinska Institutet, Stockholm, Sweden; 11 Department of Laboratory Medicine, Karolinska Institutet, Stockholm, Sweden; 12 Department of Medical Biochemistry and Biophysics, Karolinska Institutet, Stockholm, Sweden; 13 Department of Neurobiology, Care Sciences and Society, Karolinska Institutet, Stockholm, Sweden; 14 Theme Inflammation and Aging, Karolinska University Hospital, Stockholm, Sweden; 15 School of Biotechnology, Chemistry and Health, Science for Life Laboratory, Royal Institute of Technology, Stockholm, Sweden; 16 Department of Neurology, Karolinska University Hospital, Stockholm, Sweden; 17 Department of Clinical Neuroscience, Karolinska Institutet, Stockholm, Sweden; 18 Department of Medicine, Karolinska Institutet, Huddinge, Stockholm, Sweden; 19 Department of Clinical Science, Intervention and Technology, Karolinska Institutet, Stockholm, Sweden

**Keywords:** Genome sequencing, Rare diseases, Clinical diagnostics, Single nucleotide variants, Chromosomal rearrangements, Structural variants, Precision medicine

## Abstract

**Background:**

As clinical genetics evolves towards the broader field of clinical genomics, the diagnostic approach to rare diseases is undergoing a paradigm shift. This transformation has significantly impacted rare disease diagnostics, increasingly done through gene panels, whole exome and whole genome sequencing. To advance beyond genomics into precision medicine and encompass the breadth of relevant clinical scenarios, a true systems shift is required that challenges conventional barriers and enables the formation of cross-disciplinary, integrated environments.

**Methods:**

The Genomic Medicine Center Karolinska Rare Diseases (GMCK-RD) has, for the past 10 years, brought together healthcare and academia to enable large-scale genome sequencing in a clinical diagnostics context. Within GMCK-RD, experts from various medical disciplines collaborate closely with clinical geneticists, bioinformaticians, and researchers to integrate genome sequencing into healthcare.

**Results:**

In total, 15 644 individuals with suspected rare diseases were analyzed using clinical genome sequencing, including pediatric (48%), adult (48%) and fetal (4%) samples. The overall diagnostic yield was 22.6%, providing a diagnosis for 3 538 individuals with variants in 1 570 genes. Moreover, a rare disease analysis tool suite developed and validated *in house* includes a bioinformatic pipeline allowing for comprehensive data analysis covering a wide range of genetic variants including SNVs, INDELs, repeat expansions, uniparental disomies, balanced and unbalanced structural variants as well as insertions of mobile elements. Results are visualized and interpreted in custom-developed decision support systems functioning as an interpretation portal as well as a knowledge-base to capture the interpretation efforts made in a structured format allowing future secondary use.

**Conclusions:**

Altogether, GMCK-RD has shifted healthcare in our region towards precision diagnostics. We emphasize the need to transition from traditional clinical genetic diagnostics to a broader clinical genomics approach. Beyond this shift, we advocate integrating genomics with specialized clinical and laboratory medicine, a concept pioneered for inborn errors of metabolism (IEM) with stepwise spread to additional disease groups. In this model, a multidisciplinary unit combines screening, targeted diagnostics, individualized treatment, and long-term patient follow-up. Here we provide a road map and guide for inspiration for centers aiming to implement genome sequencing in rare disease diagnostics.

**Supplementary Information:**

The online version contains supplementary material available at 10.1186/s13073-026-01611-3.

## Background

Healthcare is currently undergoing a transition towards precision medicine and a crucial step in enabling personalized therapies and interventions is a well-functioning workflow for precision diagnostics. This is especially relevant in the field of rare diseases where genomics analysis has revolutionized diagnostics, resulting in higher diagnostic yields with shorter turn-around times.

The two main genomic approaches used in genetic testing for rare diseases are whole exome sequencing (WES) and whole genome sequencing (WGS). Although both methods yield similar diagnostic rates of approximately 20–50% (reviewed in [1]) and perform similarly in detecting coding single nucleotide variants (SNVs) and large copy number variants (CNVs), WGS provides additional benefits [2, 3]. In particular, WGS enables the detection of non-coding variation, a broader spectrum of structural variants (SVs), short tandem repeats (STRs), and, with specialized software, some paralogous regions [4–6].

Many initiatives from across the globe are prioritizing rare diseases as one of the focus areas in their genomic medicine programs. Today, thousands of rare disease patients are assessed and analyzed by WES in a clinical setting each week. However, as more centers transition to WGS, there is considerable variability in the pipelines and workflows used, leading to differences between centers in the types of variants detected, how they are prioritized, and which findings are reported.

This article delves into the journey of Genomic Medicine Center Karolinska Rare Diseases (GMCK-RD) in the Stockholm healthcare region, highlighting its collaborative approach, methodologies, and the transformative impact on diagnosing and managing patients with rare diseases. We outline the key steps necessary for a successful integration of WGS into rare disease diagnostics – ranging from sequence generation, variant calling and prioritization to clinical interpretation and reporting, while also addressing other critical factors like data structuring, storage and reanalysis. Furthermore, we discuss the criteria for selecting patients for genome sequencing, providing specific guidelines for different disease groups. Lastly, we present a strategy for the transition from genomics into precision medicine, a long-term systems shift that necessitates deep clinical integration across multiple specialties, challenging the current organization of healthcare.

## Methods

### The Genomic Medicine Center Karolinska – Rare Diseases

GMCK-RD was initiated formally in 2017, as a node in the national Genomic Medicine Sweden initiative [7, 8], building on a collaboration that started years earlier. In brief, the initiative has four partners; the Clinical Genomics research infrastructure at SciLifeLab (Stockholm), which is responsible for sequencing, bioinformatics and data management, and three hospital-based clinical units at the Karolinska University Hospital (Clinical Genetics and Genomics (KGG), the Centre for Inherited Metabolic Diseases (CMMS), and Clinical Immunology and Transfusion Medicine (KITM)), which are responsible for setting inclusion criteria, interpreting results and clinical reporting. In the Stockholm healthcare region, diagnostic WGS for rare diseases is reimbursed within the publicly funded healthcare system. Over the study period, per-sample costs have decreased in line with sequencing technology development and consolidation of workflows. For some patient groups, such as those with intellectual disability, WGS now largely replaces combinations of earlier tests (e.g. targeted gene panels, exome sequencing and chromosomal microarrays) and is cost-comparable to previous multi-step diagnostic workups, while providing a broader range of detectable variant types. Other patient groups have not undergone systematic genetic testing prior to WGS, so its implementation in these groups likely increases costs compared with earlier, less comprehensive diagnostic pathways. On the other hand, cost savings resulting from prevention of e.g., neurological handicaps by early treatment of inborn errors of metabolism (IEM) are substantial, but these remain to be quantitated.

In addition to genomic diagnostics, the GMCK-RD initiative provides genetic counselling, family testing, and referrals for clinical surveillance, treatment, and long-term follow-up when appropriate. For many disease groups, diagnostic results are systematically discussed in regular multidisciplinary conferences (e.g. at CMMS for IEM/epilepsy and at KITM for PID/IBMFS), allowing rapid translation to individual treatment and establishment of plans for surveillance and follow-up. For some disease groups, e.g. subsets of IEM, neuromuscular disorders and primary immunodeficiencies, dedicated quality registries are available but for most rare diseases, outcome data are not systematically collected.

Over the 10-year period, GMCK-RD has grown from a small pilot collaboration to a regional service with dedicated staff across sequencing, bioinformatics and clinical interpretation. The current team includes 34 clinical geneticists, 14 other medical specialists, 18 clinical laboratory geneticists, and 15 bioinformaticians directly involved in rare disease WGS. Demand is managed through standardized referral criteria, triaging of urgent cases, and weekly multidisciplinary meetings at each clinical unit. Workforce competence is maintained through structured in-house training for new staff, regular case-based seminars, participation in external quality assessment schemes and active engagement in national working groups within Genomic Medicine Sweden [8]. The Swedish national certification process for clinical laboratory geneticists, coordinated by the Swedish Society for Medical Genetics and Genomics (SFMG), is a structured, workplace-based training programme of at least two years with defined learning objectives, culminating in a formal examination by two independent external examiners (a specialist in clinical genetics and a certified clinical laboratory geneticist) who assess that all competencies have been achieved.

GMCK-RD currently offers three distinct clinical genome pathways; two integrated precision medicine workflows, available through CMMS and KITM, and a general genetics service provided through KGG. A detailed description of the partnership is given in Stranneheim et al. [2]. Since then, the concept has been further developed by supporting multidisciplinary workflows in order to implement genomics all the way into acute clinical medicine.

## Material

### Patients

The study describes an ongoing, real-time implementation of genomics into clinical medicine across a complex healthcare system. Patients were referred from various subspecialized clinics within the Stockholm healthcare region, as well as for some disease groups, from the whole of Sweden. Some also originate from primary care and from school health physicians. Different workflows were customized to meet these highly variable clinical scenarios, as described in the results section.

All patients were referred for diagnostic WGS testing from January 1 st 2015 until December 31 st 2023. Altogether 11,274 cases were analyzed through the general genetics (KGG) arm and 3,571 and 799 cases through CMMS and KITM, respectively.

### Genome sequencing

The overall workflow for clinical WGS has been custom built by Clinical Genomics and has been described in detail previously [2]. In brief, since 2015, samples have been sequenced using PCR-free whole-genome sequencing protocols on various generations of high-throughput short-read sequencing platforms, including HiSeq X (2015–2018), NovaSeq 6000 (2018–2023) and NovaSeq X Plus (2023-) to approximately 30 × median coverage. Today, library preparation and associated sequencing is carried out four times per week, followed by automated start of bioinformatic analysis.

### Bioinformatic analysis

**Fig. 1:**
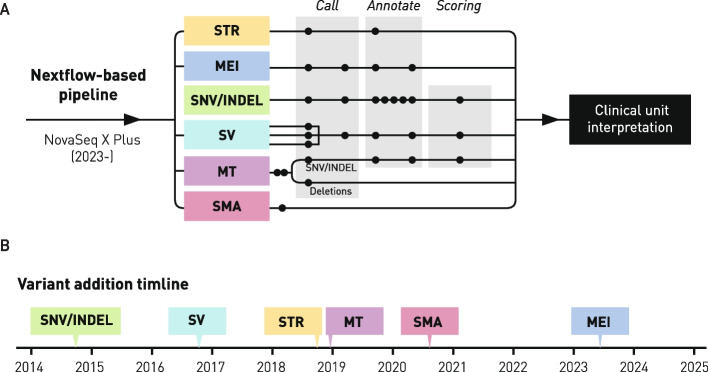
**A** Schematic description of the bioinformatic WGS analysis pipeline for rare diseases at GMCK-RD. Different callers and annotations steps are shown as black circles. **B** Timeline over when new variant types were added to the pipeline. SNV/INDEL, single nucleotide variants / insertions and deletions; SV, Structural Variants; STR, Short Tandem Repeats; MT, Mitochondrial Variants; SMA, Spinal Muscular Atrophy (SMN1 copy number analysis); MEI, Mobile Element Insertion.

The bioinformatic analysis includes calling of single nucleotide variants (SNVs), insertions and deletions (INDELs), short tandem repeats (STRs), uniparental disomies and structural variants (SVs) including deletions, duplications, inversions as well as insertions of mobile elements (MEI) [2, 9]. Also, variants in the mitochondrial genome are analyzed. Finally, the number of *SMN1* and *SMN2* copies can be estimated [4]. The bioinformatic pipelines have during the recent years been transitioned from the previously reported MIP pipeline to the current nextflow-based pipeline, which we have made publicly available on the nf-core pipeline repository (https://github.com/nf-core/raredisease) (Additional File 1: Figure S1). The current pipeline is summarized in Fig. 1.

Regions with high sequence homology and segmental duplications are challenging for short-read WGS. Currently, we routinely perform validated analyses for a subset of such loci, including *SMN1/SMN2* copy-number analysis for spinal muscular atrophy (SMA module). For other genes situated in complex paralogous regions, short read WGS is not yet used for routine testing due to limitations in sensitivity and specificity. In such cases, complementary testing using targeted assays is performed. Ongoing development aims to expand the set of clinically validated analyses in segmental duplication regions.

STR expansions are detected using ExpansionHunter [5] integrated into the nf-core/raredisease pipeline. For each locus, the tool provides an estimated repeat count, which is used to classify alleles as normal, intermediate, premutation or full expansion based on locus-specific thresholds. The WGS-based size estimate is not sufficient for clinical reporting, and all clinically relevant expansions (full mutations and pre mutations) are confirmed and more precisely sized using orthogonal methods (e.g., PCR-fragment analysis or Southern blotting) before final reporting.

### Data structures and storage infrastructure

Upon completion of the bioinformatic analysis, a data structuring process is initiated consisting of storage of selected key files; this includes raw sequence data, alignment files, raw variant files (vcf) as well as annotated variant files. The latter provides a snapshot of the information that was available upon clinical interpretation of the variants. Results of the clinical interpretation carried out in Scout [2], a custom-developed decision support system, is also captured in static reports, including dismissed variants, variant-level comments and variant classifications according to ACMG guidelines [10]. Metadata associated with sequencing and bioinformatic analysis is structured using HL7 FHIR resource GenomicStudy (https://build.fhir.org/genomicstudy.html) to facilitate interoperability and data exchange.

Reanalysis of WGS data is enabled by storage of raw files (fastq) and structured clinical interpretations. At present, reanalysis, starting from raw data, is primarily performed on a targeted basis: (i) upon clinician request when the phenotype evolves or new clinical information emerges, and (ii) when updates to relevant gene panels (e.g., new morbid OMIM or PanelApp genes) warrant reassessment of previously unsolved cases at multidisciplinary rounds, particularly within the CMMS and KITM workflows. Systematic time-based reanalysis (e.g., at fixed 2–3-year intervals) for unsolved rare disease cases is not yet implemented but is a planned future development. Nevertheless, European recommendations state that laboratories are not expected to re-analyze data systematically and report novel findings unless explicitly requested or conducted as part of quality assurance activities [11].

### Computational prioritization of called variants

**Fig. 2:**
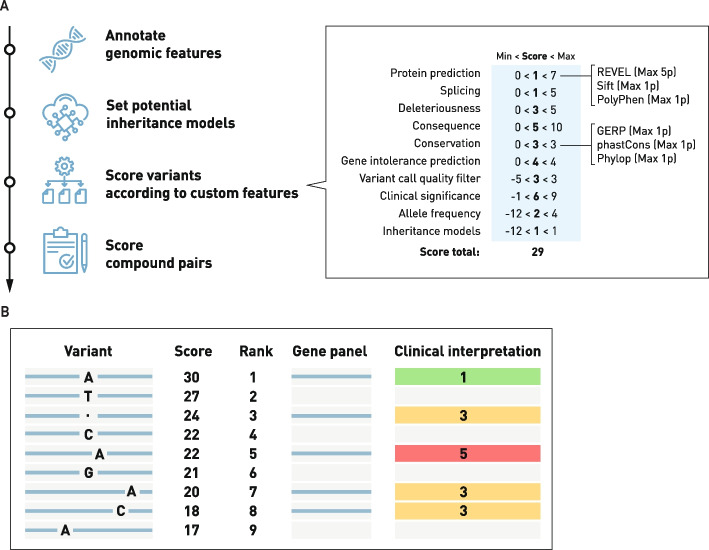
**A** Schematic of the different variant annotation steps that start with genomic features, inheritance model, scoring based on multiple parameters and finally scoring of compound pairs. To the right the specific scored paraments that are shown with the example variant reaching a rank score of 29. **B** Top-ranked variants are further assessed and scored according to the ACMG criteria with Class 4 and 5 reported as pathogenic. Green = Class 1 - Benign, Yellow = Class 3 – Uncertain significance, Red = Class 5 – Pathogenic

Called variants are annotated and prioritized using an in-house developed pathogenicity scoring system Genmod [2, 12] (Additional File 1: Figure S2). This system assigns a rank score to each variant based on multiple parameters, including functional impact, inheritance model, allele frequency, and presence of a second allele for compound heterozygosity (Fig. 2A). Of note, variants classified as Likely Pathogenic or Pathogenic in ClinVar, with a gold star review status, are always retained regardless of frequency or consequence.

To evaluate the performance of this prioritization approach, we retrospectively analyzed 3,042 previously reported pathogenic variants. For each case, the relevant version of the rank model was applied, followed by filtering using the appropriate gene panel and the Scout clinical filter. This filter retains variants with moderate to high predicted impact (based on VEP [13]), located in exonic or splice-site regions, and below strict population allele frequency thresholds (gnomAD [25] < 1% for SNVs and INDELs; < 1% in the local count database for SVs).

Rank model versions with minor updates, such as changes in label nomenclature or local database expansion, were grouped for consistency, and structural variants were evaluated in parallel using dedicated SV-specific rank groups.

### Interpretation of called variants

Following bioinformatic analysis and variant ranking, the data from individual cases are interpreted at the clinical unit responsible for the patient's case. To limit the number of variants that warrant manual assessment, the above-mentioned Scout clinical filter is applied. The ranked variants are then further filtered based on phenotype and inheritance (Fig. 2).

#### Phenotype-based filtering

The phenotype-based strategy relies on in silico gene panels (referred to as panels) and restricts the clinical interpretation to variants in genes associated with the patient's phenotype and disease. GMCK-RD panels are regularly updated with new disease genes according to PanelApp [14], OMIM (https://omim.org/), targeted PubMed searches, personal communication with experts (such as European Reference Networks and other national and international expert groups) as well as presentations at conferences. Personalized gene panels may also be created upon request for analysis of specific genes based on clinical indication or when no established panel is suitable. In such cases, patient-specific Human Phenotype Ontology (HPO) [15] terms help build custom panels, or suitable PanelApp panels can be adapted.

#### Inheritance-based filtering

The inheritance-based strategy is used when family members have been included in the analysis and employs a genotype-driven approach, filtering for variants that are heterozygous, bi-allelic, or X-linked. In family-based analyses, it is possible to filter based on inheritance patterns, for example: compound heterozygous recessive, de novo dominant, and multigenerational dominant. Of note, variant ranking is also influenced by inheritance (Fig. 2A).

#### Variant interpretation

Variant interpretation is conducted in Scout [2], a purpose-built decision support system. The platform presents each case with an overview of chromosome coverage using ideograms and separate tabs for querying SNVs/INDELs, structural variants, repeat expansions, and mobile element insertions. Custom filters can be applied to each category, and variants can be annotated, ACMG-classified, and pinned for follow-up. The system integrates data from publicly available population frequency databases (gnomAD [16], SweFreq [17]), our in-house frequency database (LocusDB [18]), ClinVar [19] and in silico pathogenicity tools (SIFT [20], Polyphen 2 [21], CADD [22], REVEL [23]) and provides various other annotations (Fig. 2). Variants previously reported as pathogenic are flagged and known founder variants are also highlighted. When warranted, in addition to the Scout evaluation, a digital chromosome analysis is performed using vcf2cytosure [6], which enables a genome-wide analysis that simulates chromosomal microarray analysis (CMA). This method allows data to be processed within the same system as clinical arrays, enabling detected variants to be annotated and compared to the in-house database from ~ 10 000 cases previously analyzed by CMA.

The case assessment starts with a specially trained clinical laboratory geneticist who reviews the data and flags variants of interest. Variants are evaluated based on Scout rank scores and additional information from updated population databases (gnomAD [16]) and ClinVar [19]. Of note, if a variant is reported as pathogenic in ClinVar, it is highlighted in the interpretation system.

Different allele frequency thresholds are applied to discard variants depending on the suspected inheritance pattern (dominant or recessive), the expected penetrance and age of onset. For dominant pediatric diseases with an early onset and high penetrance, variants are discarded if they are present more than five times in gnomAD v2. In contrast, the most common variant reported, a risk allele for inherited breast cancer in *CHEK2,* is present in 1/240 individuals in gnomAD v2. In the case of recessive disorders, variants are discarded if more than five individuals are homozygous for the variant in gnomAD v2. This is likely too high and has been complemented over time with panel specific criteria based on age of onset and penetrance.

In addition to canonical splice variants, we also investigate potential splice-altering variants within the intronic regions (± 20 bases). Deep intronic variants already reported in ClinVar are also considered, as discussed above. For gene-specific phenotypes involving a limited number of genes (1–2 genes), we additionally assess all rare (gnomAD AF > 0.001) intronic variants. Possible effects on splicing are further evaluated using bioinformatics tools such as splice prediction software in Scout (SPIDEX [24], SpliceAI [25]) as well as software incorporated in Alamut VisualPlus (Sophia Genetics). Follow up evaluation is done with RNA analysis using cDNA sequencing or whole transcriptome sequencing.

This genetic assessment, based on a joint evaluation of all these factors results in a short list of variants that undergo medical evaluation. The medical doctor(s) evaluates the selected variants in concordance with the detailed patient symptoms and variants deemed as potentially disease causing are reported. At this step, the three clinical workflows in GMCK-RD diverge and the specific multidisciplinary teams have created tailored criteria for inclusion as well as recommendations for WGS data interpretation, ensuring that reported variants are directly relevant and beneficial for the needs of the patients and their family members. Importantly, data can be shared between teams, enabling broad as well as targeted analyses in patients with unclear, atypical and evolving clinical phenotypes.

### Pre-test counselling and incidental findings

Pre-test counselling is provided by clinical geneticists or physicians with training in genomic medicine. In line with European guidance, which differs from some non-European practices, we do not actively search for secondary findings unrelated to the indication for testing (e.g. ACMG ‘secondary finding’ lists) and restrict primary analysis to genes considered relevant to the clinical question [26]. Nevertheless, incidental findings occasionally occur, most commonly in cancer predisposition or cardiac genes that are part of panels for recessive disorders or overlapping phenotypes. In such cases, we follow a structured process similar to that described for the IC panel below: the patient’s personal and family history, expected variant penetrance, and available preventive measures are carefully considered, and an individualized decision is made on whether to report the finding. Sex chromosome aneuploidies or other clearly pathogenic findings discovered are reported when they are deemed clinically relevant for the patient.

### General genetics WGS workflow at Clinical Genetics and Genomics (KGG)

In the general genetics workflow for clinical WGS at the Department of Clinical Genetics and Genomics, all referrals are reviewed by a medical doctor (either a clinical genetics specialist or a supervised resident). Based on the information provided in the referral, and when needed, additional information from the patient’s electronic health record, the physician determines which gene panel should be applied.

Within this framework, 11 274 WGS analyses were performed over a nine-year period, from 2015 to 2023. Of these, 1 126 (10%) were analyzed using a family-based approach, while 6 853 (61%) underwent singleton analysis with one of the seven most frequently used phenotype-based panels. The remaining 3 295 analyses (29%) were assessed using either smaller curated panels or custom-made panels tailored to the individual’s clinical presentation.

For individuals with highly specific symptoms, curated panels are available for conditions such as disorders of sex development, retinopathies, hearing loss, and ciliopathies. In many of these cases, patient-specific gene panels are also generated based on phenotype data using Human Phenotype Ontology (HPO) terms [15] or tools like PanelApp [14].

Below, we describe the clinical indications for the seven most commonly used panels, including associated symptoms, diagnostic criteria, and relevant coexisting conditions that support the use of WGS and guide panel selection. We also provide an overview of the family-based approach and prenatal testing using genome sequencing.

#### Intellectual disability (ID panel)

The ID panel is used for a heterogeneous group of individuals with neurodevelopmental disorders (NDDs), including intellectual disability (ID), autism spectrum disorder, developmental delay, speech and language disorders and related phenotypes. In many cases, a specific subdiagnosis within the NDD spectrum had not yet been established at the time of genetic testing. Additional clinical features are sometimes present alongside NDD. The vast majority of referred cases (90%) were children (average 9 years; range 0–58). The gender distribution was 63% male and 37% female.

The ID panel includes 1,567 genes and STRs are assessed at seven loci (Additional File 2: Table S1). The genetic analysis also incorporates genome-wide detection of structural variants using a pipeline that replaces the need for CMA, referred to as WGS-CMA. Due to the large number of genes included, the analysis primarily focuses on clearly pathogenic variants (ACMG class 4 and 5). Variants of uncertain clinical significance (VUS) are generally not reported; however, VUS deemed clinically relevant, such as those with strong gene-disease association and phenotype overlap, may be included in the clinical report after multidisciplinary review. In cases where an uncertain variant warrants further investigation, the result is classified as inconclusive and parental samples are requested for Isegregation analysis.

#### Neuromuscular, ataxia and spastic paraplegia disorders (NMD panel)

The NMD panel is used for a clinically heterogeneous group of individuals with suspected neuromuscular disorders (NMDs), including myopathies, neuropathies, spastic paraplegia, and ataxia. The gender distribution was 54% male and 46% female. The age of the referred individuals spanned from infancy (0–1 years, 11%), childhood (2–17 years, 23%), and adulthood (18–69 years, 56%) to more than 70 years of age (10%). Regardless of age, all samples in the NMD panel were analyzed as singletons.

Most individuals have a clinical diagnosis of myopathy or neuropathy prior to genetic testing. However, cases with nonspecific neuromuscular symptoms, such as hypotonia or arthrogryposis, are also tested using the NMD panel as part of a broader diagnostic evaluation. The panel also includes individuals with overlapping phenotypes, such as ataxia, that may fall within the neuromuscular spectrum. The NMD panel includes 1,035 genes (Additional File 2: Table S2), covering established NMD genes as well as those associated with spastic paraparesis, ataxia, and other movement disorders. The analysis includes STR-analysis for 29 loci (Additional File 2: Table S2) and a specific assessment of the *SMN1*-gene copy number. As with other large panels, the reporting of VUS is minimized and restricted to variants with potential clinical significance. When available, clinical findings and results from neurophysiological investigations, muscle biopsy analyses, biochemical analyses are used to support the interpretation. Parental samples are often difficult to obtain for adult individuals, but in pediatric cases, parental testing may aid in the variant interpretation.

#### Inherited cancer (IC panel)

The IC panel is used for individuals with a suspected hereditary cancer predisposition and includes both children (37%) and adults (63%) under different inclusion criteria. For children, all newly diagnosed cancer cases in Sweden have been part of a national study using paired genome sequencing (tumor/normal) since 2021 [23] and are sequenced as part of clinical routine since 2024. Although initially conducted as research, these samples are processed through the clinical laboratory, and cancer predisposition variants are reported. Adult individuals are referred for testing based on family or personal medical history suggestive of a genetic predisposition, such as early-onset cancer, multiple primary tumors, or suspected hereditary cancer syndromes.

The IC panel consists of 165 genes (Additional File 2: Table S3), covering a broad range of hereditary cancer conditions, from genetically heterogeneous conditions like hereditary paraganglioma to single-gene disorders such as retinoblastoma. Analysis is conducted on a singleton basis. Variants identified in the IC panel are reported following gene-specific ACMG criteria for pathogenicity (such as *TP53* [27] and *CDH1* [28]). Risk factors for cancer predisposition are reported only when national care guidelines exist. Examples of cases where a risk variant would lead to special surveillance include families with hereditary breast cancer where truncating variants in *ATM, BARD1, CHEK2, RAD51C* and *RAD51D* result in annual mammograms instead of every two years and families with hereditary ovarian cancer where carriers of truncating variants in *BRIP1, RAD51C* and *RAD51D* are offered an option of post-menopausal salpingo-oophorectomy. VUS are rarely reported. If the variant is a possible de novo variant in a child with cancer, the analysis is reported as inconclusive and parental samples are requested before a final report is issued. Of note, we do not report out carriership of heterozygous missense variants in genes that cause autosomal recessive conditions, such as *MUTYH or RAD51C,* as carriership does not lead to an increased risk of cancer that would lead to additional surveillance of the patient.

Cancer predisposition variants may also be incidentally detected during WGS analysis for other indications, such as truncating variants in *BRCA1/2* or *PALB2* which are present in 0.2% of the gnomAD population [16]. Even though the targeted analysis approach minimizes the risk of incidental findings, some cancer genes are included in other gene panels as they can also cause autosomal recessive disease. For instance, bi-allelic *ATM* variants can cause autosomal recessive ataxia-telangiectasia and bi-allelic *BRCA2* can cause autosomal recessive Fanconi anemia and can thus be detected by chance. If this happens, a comprehensive review of the patient’s medical- and family history is conducted, and an individualized decision on whether to report the variant as an incidental finding or not is made for each patient based on the ethical principles that guide health care as well as what is known about the variant, the expected penetrance, the availability of prevention programs and the family history.

#### Connective tissue disease (CTD panel)

The CTD panel is used for individuals with suspected heritable connective tissue disorders (HDCTs) and heritable thoracic aortic disease, including conditions such as Marfan syndrome, Loeys-Dietz syndrome and Ehlers-Danlos syndrome. The majority of the referrals were adults (81%) with children accounting for 19%. All samples were analyzed as singletons.

The CTD panel includes in total 154 genes (Additional File 2: Table S4). In addition to HDCT-related genes, it also covers genes relevant for differential diagnoses due to overlapping clinical features with other inherited conditions, such as mild skeletal dysplasias, collagen-related myopathies, and Birt-Hogg-Dubé syndrome.

Typically, variants classified as disease causing (ACMG Class 4 and 5) are reported. However, VUS are sometimes included in reports.

#### Neurodegenerative disorders (NeuroDeg panel)

The NeuroDeg panel is used for individuals with suspected adult-onset neurodegenerative conditions. The majority of referrals (> 80%) involved individuals with clinical suspicion of dementia or other neurodegenerative disease. All tested individuals were adults, with 88% over the age of 50 years. All samples were analyzed as singletons.

The NeuroDeg panel consists of 138 genes and includes STR analysis at 17 loci (Additional File 2: Table S5). It targets disorders associated with cognitive impairment and dementia, such as Alzheimer disease, frontotemporal dementia, and dementia with Lewy bodies, as well as causative genes for Creutzfeldt-Jakob disease, ALS, and Parkinson’s disease. The known mutation spectrum across these genes is highly diverse encompassing a wide range of variants, from large duplications (e.g., *APP* [29, 30]) to SNVs, STRs, and small deletions (e.g., *GRN*). Many neurodegenerative diseases exhibit overlapping clinical presentations, making diagnostics challenging. However, identifying a causative genetic variant can establish a definitive diagnosis, eliminating the need for a postmortem neuropathological examination.

Typically, variants classified as disease causing (ACMG Class 4 and 5) are reported. However, VUS are sometimes included in reports.

#### Skeletal dysplasia disorders (SKD panel)

The SKD panel is used for a heterogenous group of patients with suspected genetic skeletal disorders, based on clinical and radiographic findings of abnormal skeletal morphology or abnormal bone density. The majority of referrals were children (59%), with adults accounting for 30% and prenatal samples for 11%. All samples were analyzed as singletons. Before genetic testing is initiated, basic clinical information is required including radiographic findings, as well as symptoms from internal organs.

The SKD panel includes 681 genes (Additional File 2: Table S6). VUSes are selectively reported only when the radiological phenotype is specific and aligns with the disorder linked to the gene in question. In cases where a VUS requires further investigation, the result is reported as inconclusive and parental samples are requested.

#### Inherited cardiac conditions (ICC panel)

The ICC panel is used for individuals with suspected inherited arrhythmias and cardiomyopathies, with a focus on early diagnosis to prevent severe complications, including sudden cardiac death. The majority of referrals were adults (88%). All samples were analyzed as singletons.

When genetic testing is initiated, detailed clinical information is required to classify individuals into defined phenotype groups, including hypertrophic cardiomyopathy, dilated cardiomyopathy, arrhythmogenic right ventricular cardiomyopathy, left ventricular non-compaction cardiomyopathy, long QT syndrome, catecholaminergic polymorphic ventricular tachycardia and Brugada syndrome. The clinical workflow is carried out in close collaboration with cardiologists.

The panel includes 94 genes (Additional File 2: Table S7) associated with both channelopathies and cardiomyopathies, including key genes for hypertrophic and dilated cardiomyopathy, long QT syndrome, and related conditions. Variants classified as disease causing (ACMG Class 4 and 5) are reported and selected VUS are included when they warrant follow-up through re-evaluation or segregation analysis. We follow the 2023 ESC Guidelines for the management of cardiomyopathies and other cardiac conditions [31, 32] and apply an evidence-based assessment of gene-disease relationships, including recent expert curation efforts in dilated cardiomyopathy [33] and hypertrophic cardiomyopathy [34] to guide gene inclusion and variant interpretation.

#### Family-based (TRIO) analysis

For highly heterogeneous disorders, a family-centered approach, typically trio analysis involving the affected individual and both parents, was often employed. This strategy is particularly valuable in pediatric cases with suspected congenital syndromes and may be used either as a first-tier test or as a follow-up after singleton panel testing, most commonly following the ID, NMD, or SKD panels. The majority of cases were children, with 13% under 1 year and 76% under 18 years of age.

The analysis includes all genes with a known association to disease based on the morbid OMIM gene list [35] and the NHS Genomic Medicine Service Signed Off panel. These gene lists are updated quarterly to reflect the most current set of genes known to cause monogenic disease. Pre-test counseling is provided by clinical geneticists or physicians with specialized training and includes discussion of the potential for incidental findings with both the affected individual and their parents.

Variant interpretation includes all variant types and considers different monogenic inheritance patterns, including de novo variants, X-linked inheritance, and autosomal recessive inheritance. The analysis also incorporates genome-wide structural variant detection using WGS-CMA, provided that the analysis had not been previously performed.

#### Fetal samples

WGS was offered prenatally in cases of suspected fetal malformations [36, 37], either as trio analysis (*n* = 148) or as singleton analysis in specific scenarios such as non-immune hydrops fetalis (*n* = 67) or suspected skeletal dysplasia (*n* = 57). The clinical workflow is carried out in close collaboration with ultrasound and fetal medicine specialists. Referral forms must clearly specify the malformations detected and indicate whether a termination is planned or if the parents are awaiting genetic results to support decision-making.

For non-immune hydrops fetalis, a targeted gene panel of 343 genes is analyzed (Additional File 2: Table S8). In fetuses with malformations, the analysis includes all morbid OMIM genes, all variant types, and genome-wide structural variant detection using WGS-CMA. Only pathogenic and likely pathogenic variants (ACMG class 4 and 5) expected to cause the condition are reported.

### Integrated WGS workflow at the Centre for Inherited Metabolic Diseases (CMMS)

The CMMS has adopted a multidisciplinary, patient-centered organizational structure combining clinical and laboratory medicine. In this unit, experienced specialists in pediatric and adult neurology, metabolic medicine, endocrinology, clinical genetics and clinical chemistry work closely together with experts in molecular genetics, analytical chemistry and bioinformatics. In all, this enables a targeted analysis of relevant genes/gene panels, integration of genome data with biochemical and clinical investigations, functional validation of unclear genetic variants and rapid translation into individualized treatment.

During the study period, 3,571 individuals underwent WGS analysis at CMMS. Indications could be broadly divided into five clinical groups where the two most common indications were suspected inborn errors of metabolism (1,859 cases; 52%) and epilepsy (774 cases; 22%). All investigations are jointly reviewed by both a clinical laboratory geneticist and an experienced senior consultant with expertise in a relevant clinical specialty. The genomic analysis includes evaluation of small-scale variants (SNVs, INDELs, SVs) as well as large-scale SVs. At CMMS, all cases are presented at multidisciplinary rounds where variants of potential interest are selected for in-depth discussion.

#### Inborn errors of metabolism including mitochondrial disorders (IEM panel)

The IEM panel is used for individuals with suspected inborn errors of metabolism (IEM), a diverse group of disorders affecting various parts of intermediary metabolism including dysfunction in the cellular organelles such as mitochondria, lysosomes and peroxisomes.

Biochemical analysis of plasma and/or urine can often detect the accumulation of molecules from intermediary metabolism and is therefore used in the diagnostic workup. Individuals with IEMs often have symptoms already in the neonatal period or later in childhood. However, patients with milder forms of these disorders may present in adulthood. Until the end of 2023, 1 091 children and 768 adults were analyzed. Samples are usually analyzed as singletons.

The IEM panel includes 1 099 genes (Additional File 2: Table S9), with most conditions inherited in an autosomal recessive manner. Genetic findings are interpreted in combination with biochemical data, which often helps confirm or exclude the relevance of uncertain variants. Some of the mitochondrial disorders are caused by variants in the mitochondrial DNA (mtDNA), and if such a disorder is clinically or biochemically suspected mtDNA is analyzed. The pool of mtDNA consists of inherited polymorphisms and, in some cases, disease-causing variants present in varying ratios across different tissues, a phenomenon referred to as heteroplasmy. In suspected mitochondrial diseases, a muscle biopsy is performed for biochemical evaluation of the respiratory chain [38]. As heteroplasmy levels are typically lower in blood-derived DNA, muscle-derived DNA is used for WGS in these cases. Variants classified as pathogenic or likely pathogenic (ACMG class 4 and 5) are reported. Class 3 variants (VUS) may also be reported when supported by relevant biochemical evidence or a strong clinical phenotype.

#### Diagnostic validation in newborn screening for inherited metabolic diseases (NBS-M panel)

The national newborn screening, primarily based on tandem mass spectrometry (MS/MS) analysis of metabolites extracted from dried filter papers or enzyme assay, is centralized to CMMS for all infants born in Sweden (about 100 000 children/year). CMMS is also responsible for the biochemical and genetic confirmation of the metabolic diagnoses for over half of the newborns. If the biochemical diagnostic analysis confirms the screening result, a genetic diagnosis can be established in nearly 100% of cases. If a single gene is responsible for the disorder Sanger-based sequencing is used for time and cost efficacy. This applies to phenylketonuria (PKU), medium-chain acyl-coenzyme A dehydrogenase deficiency (MCADD), or very long-chain acyl-coenzyme A dehydrogenase deficiency (VLCADD). For other disorders where multiple potential causative genes exist, a singleton WGS-based strategy is used. Examples of such disorders include maple syrup urine disease (MSUD) or multiple acyl-coenzyme A dehydrogenase deficiency (MADD). In cases where follow-up biochemical analysis is essentially normal, NBS-M panel analysis may be performed to exclude the suspected disease with greater certainty, thereby eliminating the need for further clinical follow-up. The panel contains 51 genes (Additional File 2: Table S10). Since the NBS-M panel started in 2021 the total number of individuals analyzed is 23.

#### Monogenic diabetes (DIAB panel)

The DIAB panel is used for individuals with suspected monogenic diabetes mellitus, a group of conditions that account for approximately 2–5% of diabetes cases diagnosed before the age of 35. The DIAB panel is typically used in the diagnostic work-up of patients whose clinical presentation is not typical of classical type 1 or type 2 diabetes, typically relatively young, non-obese individuals without pancreatic islet antibodies, often with first-degree relatives exhibiting a similar phenotype. The panel includes 54 genes (Additional File 2: Table S11) and also assesses mtDNA, with a particular focus on the *m.3243A* > *G* variant associated with maternally inherited diabetes and deafness. A total of 154 individuals have been analyzed, of which 29% are pediatric. All cases are discussed in a multidisciplinary conference, to which diabetologists from all hospitals in Stockholm are invited. Based on the specific gene identified, a tailored treatment regimen can often be implemented.

The majority of monogenic diabetes conditions follow an autosomal dominant inheritance pattern. When a pathogenic variant is identified, genetic testing is recommended for affected relatives, and a gene-specific treatment strategy can often be implemented.

#### Acute liver failure and cholestasis in children (PEDHEP panel)

The PEDHEP panel is used primarily in the evaluation of children with various types of liver disease, ranging from acute liver failure to cholestatic diseases, suspected bile acid synthesis disorders, and ductal plate malformations. The panel includes 172 genes (Additional File 2: Table S12), and the samples are analyzed as singletons. Many inborn errors of metabolism (e.g., glycogen storage disorders, tyrosinemia, and congenital disorders of glycosylation) present with a pronounced hepatic phenotype. Depending on the clinical history and biochemical work-up results, the IEM panel may be added to the genetic evaluation. Up to 2024, 233 individuals were analyzed, of whom 82% are pediatric. All cases are reviewed at a multidisciplinary conference including experts in pediatric hepatology.

#### Epilepsy (EP panel)

The EP panel is used in the evaluation of individuals with suspected genetic epilepsy, particularly those with treatment-refractory epilepsy. It is used in both adult (27%) and pediatric (73%) individuals, but the diagnostic yield is higher in pediatric cases [39]. This is likely due to the more polygenic nature of adult epilepsy and the lower rate of testing in that population. The EP panel includes 565 genes (Additional File 2: Table S13), and for young children it is often combined with IEM, as the clinical picture may be less clear in infants. The analysis is preferably performed as a trio due to the high prevalence of de novo variants.

Variant interpretation integrates detailed phenotypic characterization with paraclinical data such as electroencephalogram (EEG) and brain magnetic resonance imaging (MRI). A deep understanding of epileptology is critical for accurate interpretation. Extra precaution is warranted, as many epilepsy-related genes display reduced penetrance. Additionally, VUSes are often reported, since they may have clinical implications in the choice of anti-seizure medication. Likewise, a clinical response to a specific treatment may strengthen a genetic finding.

### Integrated WGS workflow at Clinical Immunology and Transfusion Medicine (KITM)

The KITM unit applies a multidisciplinary, patient-centered approach that integrates clinical and laboratory expertise. Referrals are assessed by medical doctors or certified clinical laboratory geneticists who determine the most appropriate gene panel based on the information in the referral. When necessary, general electronic health records are reviewed and referring physicians are contacted for additional clinical information.

Variant interpretation is performed in close collaboration between clinical immunologists and clinical laboratory geneticists, and is supported by immunological phenotyping, functional assays and other relevant laboratory data. All cases are discussed at weekly multidisciplinary rounds before results are reported back to the clinicians. Additional laboratory investigations to functionally assess variants are conducted either within the Clinical Immunology unit or in collaboration with specialized research laboratories. National multidisciplinary patient conferences are held regularly together with immunodeficiency and hematology specialists to discuss the genetic findings, guide further diagnostics testing and inform patient treatment. Three gene panels are used in this setting: Primary Immunodeficiency (PID), Autoinflammation (AID; introduced in 2023) and Inherited Bone Marrow Failure Syndromes (IBFMS) and rare hematologic conditions.

To date, 799 individuals have been analyzed, the majority of which (621 cases; 78%) were assessed using the PID panel.

#### Primary immunodeficiency (PID panel)

The PID panel is used in the evaluation of individuals with a wide spectrum of suspected immunological disorders, ranging from those with specific, well described immunodeficiencies such as severe combined immunodeficiency (SCID) to less well-defined diagnosis, such as immune dysregulation or recurrent infections. The panel is usually performed as a singleton and currently includes 482 genes (Additional File 2: Table S14). Individuals tested with this panel range from infants and children with severe early-onset phenotypes to children and adults with milder, late-onset presentations. Of the individuals analyzed, 64% were children (< 18 years) and 3% were referred through the national newborn SCID screening program, which has been active since August 2019.

#### Autoinflammation (AID panel)

The AID panel, introduced in 2023 as a smaller version of the PID panel, focusing on 73 genes (Additional File 2: Table S15) associated with the innate immune system disorders that lead to autoinflammation. This includes genes such as *MEFV*, for familial mediterranean fever. The narrower scope of the AID panel allows for more time-efficient analysis and reduces the risk of incidental findings. It is particularly suited for individuals presenting with isolated autoinflammatory symptoms without additional signs of immunodeficiency.

#### Inherited bone marrow failure (IBMFS) and rare hematologic condition panel

The panel is used for individuals with suspected bone marrow failure or rare hematologic conditions resulting in peripheral blood cytopenias, including but not limited to Fanconi anemia, telomere biology disorders, congenital neutropenia, Diamond Blackfan anemia and macrothrombocytopenia. The panel is usually performed as a singleton and currently includes 236 genes (Additional File 2: Table S16). Most individuals tested are children (82%), and commonly the analysis is conducted in parallel to chromosomal breakage and telomere length analysis.

## Results

### Turnaround time

The median turnaround time (TAT) for sequence generation and bioinformatic analysis has steadily decreased over the years and in 2023 it was 11 days for regular priority samples and 9 days for priority samples (Table 1). Express samples are rarely handled but the current TAT is about 4 days.

**Table 1:** Average turnaround times

	2017	2018	2019	2020	2021	2022	2023
Sequencing (Days)							
Standard	14.4	13.3	14.9	15.0	12.6	12.4	11.1
Priority	10.6	10.6	13.9	12.5	10.1	11.6	9.4
Total TAT (Months/Days)							
Standard	6.2/205	4.9/164	3.8/133	2.6/93	3.1/110	2.6/94	2.6/94

The total TAT from blood draw to signed clinical report is longer, as it includes additional time for sample logistics, DNA extraction, data analysis, clinical interpretation and validation with an orthogonal method when warranted. For priority and express cases, this additional time is minimized, typically adding only 2–3 days. Across the entire cohort, 73% of cases are reported within 3 months, 19% within 6 months, 6% within 1 year, and 2% after more than 1 year. The longest TATs were observed in the early years of the program, and for routine samples the TAT has steadily decreased over time, with no cases exceeding 6 months in 2024.

### Overall diagnostic findings

The overall diagnostic yield was 22.6%, resulting in a genetic diagnosis for 3,538 individuals. In total, 4,460 variants were reported across 1,570 unique genes, including 130 short tandem repeat (STR) expansions in 15 different genes (Additional File 2: Table S17). Regarding structural variants: 105 SVs were identified using the analyzed gene panels and 57 additional SVs were detected through genome-wide analysis using WGS-CMA (Additional File 2: Table S18). An overview of results across 20 different gene panels is shown in Table 2.

**Table 2:** Overview of findings from clinical genome sequencing

Team	Panels	Cases	Male	Pediatric	Pathogenic variant	VUS
KGG	ID	1495	63%	90%	22%	15%
	NMD	1428	55%	33%	24%	15%
	IC	1350	46%	37%	13%	3%
	CTD	993	45%	19%	9%	11%
	NeuroDeg	609	48%	0%	12%	5%
	SKD	520	44%	59%	43%	11%
	ICC	458	59%	0%	24%	6%
	General genetics HPO	3295	50%	53%	25%	10%
	Trio analysis	1126	50%	76%	24%	12%
CMMS	IEM	1859	54%	59%	26%	0%
	EP	774	56%	73%	26%	1%
	NBS-M	23	48%	96%	65%	0%
	DIAB	154	47%	29%	23%	2%
	PEDHEP	233	53%	82%	25%	1%
	mtDNA	390	53%	50%	7%	0%
	CMMS HPO	138	46%	59%	26%	4%
KITM	PID	621	52%	59%	30%*	0%
	IBMFS	144	51%	83%	29%*	0%
	AID	18	56%	50%	11%*	0%
	PID + IBMFS	16	53%	47%	19%*	0%

*VUS* Variant of Unknown Significance, *KGG* Clinical Genetics and Genomics, *CMMS* Centre for Inherited Metabolic Diseases, *KITM* Clinical Immunology and Transfusion Medicine, *ID* Intellectual Disability, *NMD* Neuromuscular Disorder, *IC* Inherited Cancer, *CTD* Connective Tissue Disease, *Neurodeg* Neurodegenerative Disorder, *SKD* Skeletal Dysplasia, *ICC* Inherited Cardiac Condition, *HPO* Human Phenotype Ontology, *IEM* Inherited Metabolic Disorders, *EP* Epilepsy, *NBS-M* Newborn Screening Metabolic, *DIAB* Monogenic Diabetes, *PEDHEP* Pediatric Liver Disease, *MtDNA* Mitochondrial DNA, *PID* Primary Immunodeficiency, *AID* Autoinflammation, *IBMFS* Inherited Bone Marrow Failure Syndrome

^*^Pathogenic and VUS

**Fig. 3:**
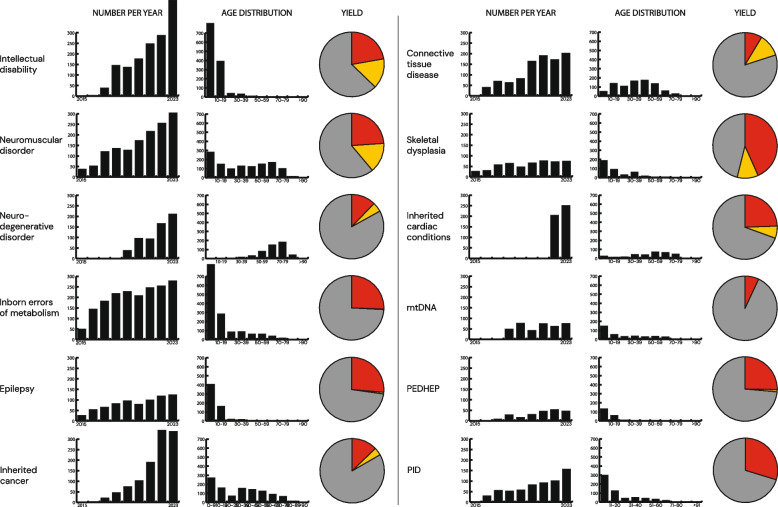
**A** Results from twelve selected gene panels including number of individuals analyzed per year, age distribution and yield (red indicates pathogenic variants (ACMG Class 5 and 4), yellow prioritized variants of uncertain significance and grey unsolved cases)

The majority (54%) of the diagnosed individuals had a pathogenic variant in a gene that was responsible for disease in only 1–3 individuals (21% (750 genes), 18% (316 genes) and 15% (180 genes) detected in one, two and three individuals respectively). A total of 31% (*n* = 1,097) of cases involved more than 10 individuals sharing the same genetic diagnosis, affecting 57 different genes. The most common findings were SNVs/INDELs in *NF1* (*n* = 59), *PTPN11* (*n* = 40), *RYR1* (*n* = 39), and *TTN* (*n* = 39). Among STRs, *C9orf72* expansion causing FTD/ALS (*n* = 39) and among SVs, the 22q11 recurrent deletion (*n* = 8) were the most common. Some of the disorders were identified by multiple teams, such as pathogenic variants in *CFTR*, *G6PD*, *NOTCH1*, *PTPN11* and *SBDS* reported by all three clinics. In addition, 225 genes were reported by two clinical teams (3 genes by CMMS and KITM, 45 genes by KITM and KGG, and 177 genes by CMMS and KGG). For the most commonly used panels, the ten most frequently reported genes are listed in Table 3, while Fig. 3 illustrates the age distribution, diagnostic yield, and the increase in the number of analyzed cases over the years. As expected, the diagnostic yield is generally higher for panels with a large proportion of pediatric cases.

**Table 3:** The top ten most commonly identified genes for ten selected panels. The gene name is followed by the number of patients in which variant(s) in this gene were identified as causative

ID		NMD		NeuroDeg		IEM		EP		CTD		SKD		ICC		PID		Trio	
*ANKRD11*	11	*RFC1 (STR)*	17	*C9orf72 (STR)*	38	*PMM2*	8	*SCN1A*	18	*FBN1*	32	*COL1A1*	23	*TTN*	26	*TNFRSF13B*	16	*ARID1B*	6
*MECP2*	9	*DMD*	16	*SOD1*	7	*OPA1*	7	*KCNQ2*	13	*COL5A1*	15	*FGFR3*	18	*MYH7*	22	*MEFV*	10	*MECP2*	5
*NF1*	7	*RYR1*	11	*SORL1*	5	*PHKA2*	7	*STXBP1*	12	*COL3A1*	13	*COL2A1*	18	*MYBPC3*	16	*CYBB*	6	*NSD1*	4
*POGZ*	7	*PMP22*	11	*TBK1*	4	*ATP7B*	6	*PRRT2*	11	*MYH11*	10	*COL1A2*	11	*KCNQ1*	13	*NFKB1*	5	*FRAS1*	4
*DDX3X*	6	*TTN*	11	*GRN*	4	*ETFDH*	6	*CACNA1A*	7	*TGFBR2*	7	*DYNC2H1*	9	*TNNI3*	6	*STAT3*	5	*HUWE1*	4
*DNMT3A*	6	*COL6A3*	9	*PSEN1*	4	*PDHA1*	6	*SLC2A1*	7	*TGFB3*	6	*EXT1*	8	*DSG2*	5	*BTK*	4	*NUS1*	3
*KMT2A*	6	*CAPN3*	8	*VCP*	3	*TPO*	6	*MECP2*	6	*COL1A1*	6	*GNAS*	7	*FLNC*	5	*ADA2*	4	*L1CAM*	3
*TCF4*	6	*SH3TC2*	6	*CHMP2B*	2	*ABCC8*	5	*SCN8A*	6	*COL2A1*	5	*COMP*	6	*PKP2*	5	*FAS*	4	*SETD5*	3
*ARID1B*	5	*MPZ*	6	*TARDBP*	2	*GALC*	5	*CDKL5*	5	*ACTA2*	5	*EXT2*	5	*TNNT2*	4	*ADA*	4	*BRAT1*	3
*FMR1 (STR)*	5	*COL6A1*	5	*ATXN8OS (STR)*	2	*GLDC*	5	*PCDH19*	5	*COL5A2*	5	*RPL13*	5	*SCN5A*	4	*FOXN1*	4	*MYH3*	3
*MED13L*	5			*MAPT*	2									*ALPK3*	4				
*PTPN11*	5			*CHCHD10*	2														
*SHANK3*	5																		

*ID* Intellectual Disability, *NMD* Neuromuscular Disorder, *Neurodeg* Neurodegenerative Disorder, *IEM* Inherited Metabolic Disorders, *EP* Epilepsy, *CTD* Connective Tissue Disease, *SKD* Skeletal Dysplasia, *ICC* Inherited Cardiac Condition, *PID* Primary Immunodeficiency

For some disease groups (e.g., IEM, epilepsy, monogenic diabetes), a diagnosis often led to changes in clinical management, such as initiation or tailoring of dietary treatment, change of antiepileptic medication, or targeted surveillance of at-risk relatives. All individuals identified with an increased risk for cancer are offered inclusion in individual surveillance programs and if applicable offered risk reducing surgery. For other disease groups, clinical utility is primarily reflected in shortening the diagnostic odyssey, ending further invasive investigations and enabling accurate genetic counselling. Long-term follow-up was beyond the scope of this study and was not quantified systematically.

### High sensitivity of variant ranking for known diagnoses

The benchmarking demonstrated that the vast majority of previously reported pathogenic variants were indeed ranked highly in our system. Out of 3,042 variants, 1,063 (35%) were ranked as the top candidate (rank 1), with a median rank position of 2 across the entire cohort of 3,042 variants. The mean rank was 5.3 ± 0.3 (95% CI, SD 8.3). Only 20 variants (0.7%) ranked below position 50 (the approximate cutoff for routine manual assessment). The maximum observed rank was 129 (Additional file 1: Figure S3).

## Discussion

Our findings show that genome sequencing is a valuable clinical test across an expanding range of disorders. As rare diseases can present across all clinical disciplines, at all ages, and range from insidious, chronic to dramatically acute diseases where rapid treatment can save lives and prevent severe handicaps, the clinical scenarios that benefit from genomic medicine are extremely variable. No single metric can capture this complexity. For example, the preferrable diagnostic yield can vary substantially depending on e.g., the heterogeneity of the clinical phenotypes and availability of treatment. We have customized our strategy to meet these variable needs, by combining genomics with classical laboratory and clinical medicine in integrated multidisciplinary workflows that differ depending on the nature of the targeted conditions. By building a format with pre- and post-test procedures customized to different clinical scenarios, we ensure that the right patients are tested for the established genes associated with their phenotype, and that test results are truly helpful in providing individualized care to the individual patient.

Obvious challenges include continuously refining the inclusion criteria and updating gene panels to accommodate the ever-growing body of genetic knowledge. As new disease groups, such as eye and hearing disorders or blood diseases (anemia, coagulation defects and erythrocyte membrane defects), start to use genomic analysis as a baseline test, extensive training and education efforts are needed. Furthermore, technological advancements like long-read sequencing [40] and RNA sequencing have shown great potential to further enhance diagnostic capability [41, 42]. Integrating these emerging technologies into clinical workflows will help refine variant interpretation and expand the range of detectable genetic alterations, which in turn will increase the diagnostic outcome.

In addition to technological advances, robust variant prioritization pipelines have been critical for enabling efficient WGS based diagnostics. In our evaluation, the majority of known causative variants ranked among the top candidates, allowing them to be rapidly identified during clinical interpretation. This high-ranking performance streamlines manual review, supports diagnostic consistency, and enhances patient safety. However, variants that received low prioritization scores frequently lacked a second allele required for compound scoring or were incorrectly penalized due to erroneous assumptions about inheritance patterns, particularly in known dominant conditions. These issues reflect known limitations of the Genmod [12] scoring model, which is currently undergoing revision. Ongoing improvements include refinement of compound scoring and integration of Bayesian models and machine learning to further optimize accuracy. Among the 20 pathogenic variants that ranked below position 50, the most common scenarios were: (i) heterozygous variant in genes that have an autosomal dominant inheritance pattern, since the ranking were initially designed to detect autosomal recessive inheritance, which lead to under-scoring of dominant or dosage-sensitive mechanisms when no other variant in the gene is present, and (ii) pathogenic variants located in exons with incomplete coverage or in regions with complex local architecture, where read-level features were down-weighted. Conversely, not all highly ranked variants were pathogenic. Many rank-1 candidates were known pathogenic variants in autosomal recessive inherited disorders, i.e. patient is a healthy carrier, where the patient’s phenotype was incompatible with the reported disease spectrum, other such variants were ultimately classified as benign or VUS after manual review, most often because the gene variant-disease association was weak, or segregation did not support causality. These observations underscore that automated ranking is a powerful triage tool but cannot replace expert clinical interpretation.

Alongside accuracy, TAT is increasingly important, particularly for acutely ill patients. The median TAT for sequencing and bioinformatic analysis has steadily decreased, reaching 11 days for routine samples and 9 days for priority cases in 2023. To offer more robust and shorter TATs, sequencing instruments are now run more frequently, aiming for 4–5 times per week in 2025, allowing for greater flexibility and faster processing. There is growing demand for genome sequencing in medically urgent scenarios, and we are developing differentiated clinical tracks targeting TATs of 2–3 days (ultra-urgent), 7–10 days (priority), and 6 weeks (routine) by 2025, calculated from reception of sample to clinical report being issued.

Of note, the total time from patient sampling to final clinical report includes not only sequencing, but also sample transport, DNA extraction and QC, bioinformatic processing, variant interpretation, and multidisciplinary review. Achieving shorter TATs across these tracks requires ongoing overall optimization of logistics, lab workflows, automation, data processing, and clinical interpretation pipelines. In routine practice, the median end-to-end time from blood sampling to signed clinical report was 2.6 months in 2023 for routine cases and significantly shorter for priority cases (< 2 weeks), reflecting both sequencing TAT and clinical interpretation time.

Access and referral patterns also influence the real-world timeliness of genomic testing. For example, individuals referred for WGS with the ID panel had a mean age of 9 years at referral. While this may reflect a substantial diagnostic delay, the cohort is clinically heterogeneous, ranging from young children with early developmental delay to individuals with mild, moderate, or severe ID, as well as those with autism, ADHD, and language delay. Referrals therefore originate from both primary and specialized pediatric care, as well as from school health physicians. Although we do not yet systematically capture socioeconomic or geographic indicators, Karolinska is the only provider of genomics-based diagnostics in the Stockholm healthcare region. The referrals to GMCK-RD originate from multiple healthcare providers from the entire Stockholm region and, for several indications, from national referral centers, which helps to mitigate inequities in access. Establishing robust inclusion criteria is an important next step. Swedish healthcare is currently going through structural changes, supporting the concept of multidisciplinary, integrated diagnostics and facilitating national coordination for selected rare disease groups. The Swedish Board of Health and Welfare coordinates a process in which selected areas are concentrated to a few units with national responsibility for highly specialized care. This will facilitate collaboration between critical expertise across the country and promote sharing of data on a national level.

Beyond clinical utility, the infrastructure we built for clinical diagnostics has also supported multiple gene-discovery projects, which are described in separate publications [43–55]. Through the GMCK-RD Matchmaker Exchange node Patient Matcher data may be shared with the wider research community [56].

**Fig. 4:**
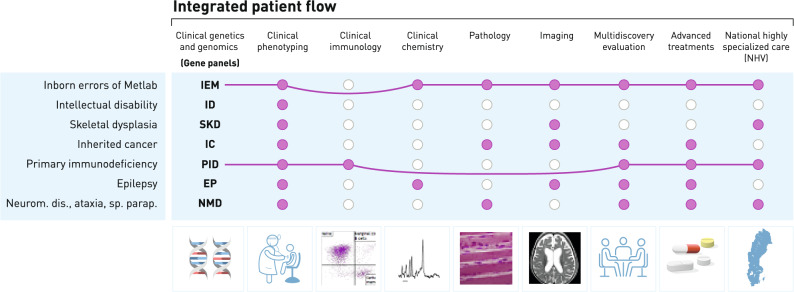
Schematic strategy for step-by-step clinical implementation of genome sequencing across multiple rare disease groups. The strategy includes both integrated patient-centered workflows and the general genetic investigation service at Clinical genetics and genomics. When fully developed, the integrated workflow concept comprises several medical specialties and organizes targeted genomics in combination with e.g., specialized functional investigations, and actively promotes national coordination. The concept is established for inborn errors of metabolism and followed by the primary immunodeficiency team, as indicated by the horizontal lines. Additional rare disease workflows, where supporting investigations are available but not organized together, are in various stages of development. National highly specialized care (NHV) in Sweden refers to the centralization of complex, rare, or resource-intensive healthcare services to a few expert hospitals to ensure high-quality and efficient care. IEM = inherited metabolic disorders, ID = intellectual disability, SKD = skeletal dysplasia, IC = inherited cancer, PID = primary immunodeficiency, EP = epilepsy, NMD = neuromuscular disorder

Implementation of genome sequencing into the management of rare diseases represents a first step in the ongoing transformation towards precision medicine. We have adopted a strategy for this gradual, long-term transition that contains two main axes (Fig. 4).

In parallel to the general genetics service transitioning from traditional tests to genome-based analysis, targeted, multidisciplinary patient flows are being developed for different disease groups using the IEM concept as a model. This is particularly important for disease groups where specialized functional investigations are essential for diagnostics and rapid treatment is critical for patient outcome. The patient flow for primary immunodeficiencies is being organized according to the principles developed for IEM, including targeted diagnostics after newborn screening for SCID and development towards a national coordination. An important challenge for the future is to establish a digital infrastructure that enables integration of data from genome analyses with other laboratory, clinical and imaging investigations to facilitate multimodal diagnostics. In parallel to the targeted workflows and equally important, the Department of Clinical Genetics and Genomics plays a pivotal role in cultivating clinical genetic expertise, serving as a hub for many teams and providing guidance in prenatal diagnostics, family investigations, and interpretation of complex genomic rearrangements. This department trains clinical geneticists and clinical laboratory geneticists who, in addition to working in the genetic service laboratory, support and/or join established and newly formed integrated teams to ensure that their expertise enhances the precision and quality of genome sequencing services. Furthermore, it guarantees that new bioinformatics pipeline modules are gradually made accessible to these integrated teams, so they can leverage the latest advancements in variant interpretation and genomic analysis. This ensures that the multidisciplinary teams remain well-informed and continue delivering high-quality, personalized care.

## Conclusions

In conclusion, we have adopted a strategy for moving from genomics into precision medicine, first by implementing genome sequencing into the clinical laboratories through GMCK-RD and followed by further implementation into healthcare through integrated multidisciplinary teams. This model is gradually consolidated and expanded as part of a long-term systems shift in rare disease diagnostics and management at Karolinska, with the aim to play a strong part in the emerging national precision medicine landscape in Sweden.

## Supplementary Information

Additional file 1: Figure S1. Steps performed in the nf-core rare disease pipeline; Figure S2. Variant scoring and prioritization with Genmod; Figure S3: Rank score performance over time.

Additional file 2: Table S1: Intellectual Disability; Table S2: Neuromuscular, ataxia and spastic paraplegia disorders; Table S3: Inherited cancer; Table S4: Connective Tissue Disease; Table S5: Neurodegenerative disorders; Table S6: Skeletal dysplasia disorders; Table S7: Inherited cardiac conditions; Table S8: Non-immune hydrops fetalis panel; Table S9: Inborn errors of metabolism including mitochondrial disorders; Table S10: Mitochondrial DNA; Table S11: Monogenic diabetes; Table S12: Acute liver failure and cholestasis in children; Table S13: Epilepsy; Table S14: Primary Immunodeficiency; Table S15: Autoinflammation; Table S16: Inherited Bone Marrow Failure; Table S17: Reported genes with diagnostic small variants; Table S18: Reported structural variants.

## Data Availability

The ethical approval did not permit sharing of WGS data, and the in-house databases used in this article are not publicly available. The nf-core pipeline is open source and available on github [57, 58]. All other softwares used, have been previously published and are cited in the text. While raw WGS data cannot be shared due to ethical restrictions, we routinely submit clinically reported variants (ACMG class 3-5) to ClinVar. All clinical gene panels used in this study are provided as Additional File 2, and updated versions are shared with the wider community on request. Novel candidate disease genes identified through GMCK-RD are reported in dedicated research articles and, when appropriate, shared via rare-disease matchmaking platforms.

## Abbreviations

ACMG: American College of Medical Genetics and Genomics
AID: Autoinflammation Diseases
CMA: Chromosome Microarray Analysis
CMMS: Centre for Inherited Metabolic Diseases
CNV: Copy Number Variant
CTD: Connective Tissue Disease
DIAB: Monogenic Diabetes
EEG: Electroencephalogram
EP: Epilepsy
GMCK-RD: The Genomic Medicine Center Karolinska Rare Diseases
HDCT: Heritable Disorder of Connective Tissue
IBMFS: Inherited Bone Marrow Failure Syndrome
IC: Inherited cancer
ICC: Inherited Cardiac Conditions
ID: Intellectual Disability
IEM: Inborn Errors of Metabolism
INDEL: Insertion and Deletion
KGG: Clinical Genetics and Genomics
KITM: Clinical Immunology and Transfusion Medicine
MADD: Multiple Acyl-coenzyme A Dehydrogenase Deficiency
MCADD: Medium-Chain Acyl-coenzyme A Dehydrogenase Deficiency
MIT: Mitochondria
mtDNA: Mitochondrial DNA
MRI: Magnetic Resonance Imaging
MS/MS: Tandem Mass Spectrometry
MSUD: Maple Syrup Urine Disease
NBS-M: Newborn Screening Metabolic
NDD: Neurodevelopmental Disorders
NeuroDeg: Neurodegenerative Disorders
NHV: National Highly Specialized Care
NMD: Neuromuscular Disorders
PEDHEP: Pediatric Liver Disease
PID: Primary Immunodeficiency
PKU: Phenylketonuria
SCID: Severe Combined Immunodeficiency
SFMG: Swedish Society for Medical Genetics and Genomics
SKD: Skeletal Dysplasia Disorder
SNV: Single Nucleotide Variant
STR: Short Tandem Repeats
SV: Structural Variant
TAT: Turnaround Time
VUS: Variant of Uncertain Clinical Significance
VLCADD: Very Long-chain Acyl-coenzyme A Dehydrogenase Deficiency
WES: Whole Exome Sequencing
WGS: Whole Genome Sequencing
