# Supplementary material for: The genomic medicine center Karolinska 10-year report on genome sequencing for rare diseases and a strategy for stepwise clinical implementation
Source: Genome Med. 2026 Mar 30;18:30. doi: 10.1186/s13073-026-01611-3 (PMC13034595; doi:10.1186/s13073-026-01611-3)
Supplement: Supplementary file 1 — Additional file 1: Figure S1. Steps performed in the nf-core rare disease pipeline; Figure S2. Variant scoring and prioritization with Genmod; Figure S3: Rank score performance over time. [file 13073_2026_1611_MOESM1_ESM.docx]

**Figure S1. Steps performed in the nf-core rare disease pipeline**

**Figure S2. Variant scoring and prioritization with Genmod**


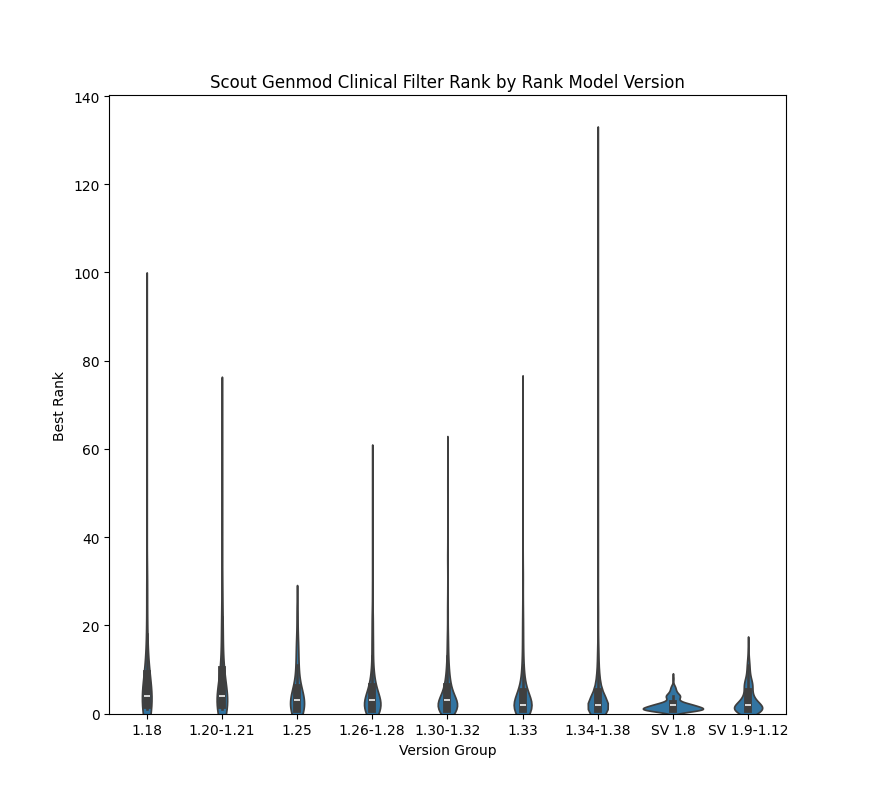


**Figure S3: Rank score performance over time**. Rank scores of 3,042 previously reported pathogenic variants across nine different versions of Genmod.
